# The bidirectional relationship between sarcopenia and disability in China: a longitudinal study from CHARLS

**DOI:** 10.3389/fpubh.2024.1309673

**Published:** 2024-05-07

**Authors:** Li Liu, Yan Zhang, Yan Shi, Lanxin Wu, Lixue Meng, Ting Zhao

**Affiliations:** School of Nursing and Health, Zhengzhou University, Zhengzhou, Henan, China

**Keywords:** sarcopenia, disability, bidirectional relationship, CHARLS, older people

## Abstract

**Objectives:**

Sarcopenia and disability represent significant concerns impacting the health of older people. This study aimed to explore the bidirectional relationship between sarcopenia and disability in Chinese older people.

**Methods:**

This study recruited older people ≥60 years old from the China Health and Retirement Longitudinal Study. In phase I, the study analyzed the relation between disability and subsequent sarcopenia using multinomial logistic regression models. Conversely, in phase II, the study assessed whether sarcopenia was associated with future disability using binary logistic regression models.

**Results:**

In phase I, 65 (16.80%) new cases of possible sarcopenia, 18 (4.65%) cases of sarcopenia, and 9 (2.33%) cases of severe sarcopenia were observed in the disabled older people and 282 (10.96%) new cases of possible sarcopenia, 97 (3.77%) cases of sarcopenia, 35 (1.36%) cases of severe sarcopenia were observed in the older people without disability. The OR (95% CI) for sarcopenia in older disabled individuals compared to those without disability was 1.61 (1.25–2.07). Adjusting for all covariates in 2011, the OR (95% CI) value for disabled individuals vs. those without disability was 1.35 (1.02–1.79). Subgroup analyses showed that disabled participants aged < 80 years were more likely to have sarcopenia (OR = 1.42, 95% CI: 1.07–1.89), and the risk of sarcopenia did not differ significantly between sex subgroups. In phase II, 114 cases (33.83%) in the possible sarcopenia patients, 85 cases (28.91%) in the sarcopenia patients, 23 cases (35.94%) in the severe sarcopenia patients, and 501 cases (16.10%) in the individuals without sarcopenia showed symptoms of disability. The OR (95% CI) for disability was 2.66 (2.08–3.40) in the possible sarcopenia patients, 2.12 (1.62–2.77) in the sarcopenia patients, and 2.92 (1.74–4.91) in the severe sarcopenia patients compared with the no sarcopenia patients. After adjusting for all covariates in 2011, the OR (95% CI) values were 2.21 (1.70–2.85) in the possible sarcopenia patients, 1.58 (1.14–2.19) in the sarcopenia patients, and 1.99 (1.14–3.49) in the severe sarcopenia patients, as compared to the older people without sarcopenia. Subgroup analyses showed that compared with men, women with possible sarcopenia had a higher risk of disability (OR = 2.80, 95% CI: 1.98–3.97). In addition, participants aged < 80 years with sarcopenia or severe sarcopenia s were more likely to have disability (OR = 2.13, 95% CI: 1.52–2.98; OR = 2.98, 95% CI: 1.60–5.54).

**Conclusion:**

The occurrence of disability increase the risk of sarcopenia in the older people, and baseline sarcopenia predicts the future disability in older people.

## Introduction

With the increasing aging of the population, the prevalence of sarcopenia is on the rise. Currently, ~50 million older persons worldwide suffer from sarcopenia, and this number is expected to reach 500 million by 2050 (1, 2). In Japan, the prevalence of sarcopenia among older individuals is 11.5–16.7% (3), while in China, it is 26.6% (4). Older people with sarcopenia experience significantly lower quality of life in terms of physical function, health status, and social function (5), and they are at a higher risk for falls, disability, death, cognitive impairment, and depression (6–12). Additionally, sarcopenia can either cause or exacerbate other conditions such as osteoporosis (13) and coronary heart disease (14). Therefore, it is crucial to identify the risk factors for sarcopenia in order to develop effective prevention programs. Furthermore, disability is also a significant issue in the aging population. In China, the number of disabled older people is projected to exceed 42 million in 2020 and reach 137 million by 2030 (15, 16). Disability negatively impacts the quality of life of the older people, and adds to the burden of care for their families and society (17). Therefore, it is important to study the factors influencing disability and work toward preventing disability in older people.

Several studies have analyzed the relationship between sarcopenia and disability, Xu et al. (7) found that sarcopenia was independently associated with disability in community-dwelling older people in China, with those suffering from sarcopenia being approximately twice as likely to be disabled in ADLs compared to those without sarcopenia. Kitamura et al. (3) demonstrated that older Japanese sarcopenia patients had an increased risk of disability, and there was no significant increase in disability risk for those with possible sarcopenia and those with only low muscle mass. However, no relevant studies have explored the interrelationship between the two. Considering the shared influencing factors and pathophysiological mechanisms between sarcopenia and disability, such as age, physical activity, inflammatory responses, and levels of oxidative stress, it is possible that they may interact with each other.

This study aims to analyze the relation between sarcopenia and disability based on the findings of the China Health and Retirement Longitudinal Study (CHARLS). In phase I, the study assessed disability and future sarcopenia's connection. In phase II, the study analyzed the relation between the presence of sarcopenia and disability.

## Methods

### Data sources

In this study, we utilized data from CHARLS (18). The data set is a longitudinal, nationally representative cohort survey with people in China aged 45 years and older, aiming at collecting information related to social, economic and health conditions. The national baseline assessment was conducted in 2011, involving ~17,000 participants, and follow-up assessments were carried out in 2013, 2015, and 2018, and there were studies described CHARLS in more detail (18, 19). The data of 2011 and 2015 were used for this study. In phase I, we focused on individuals without sarcopenia in 2011, dividing them into disability and no disability groups, and then followed up to 2015 to assess the development of possible sarcopenia, sarcopenia, and severe sarcopenia. In phase II, we studied individuals without disability in 2011, categorizing them into no sarcopenia, possible sarcopenia, sarcopenia, and severe sarcopenia groups, and followed them up to 2015 to assess disability status.

### Participants

**Figure 1:**
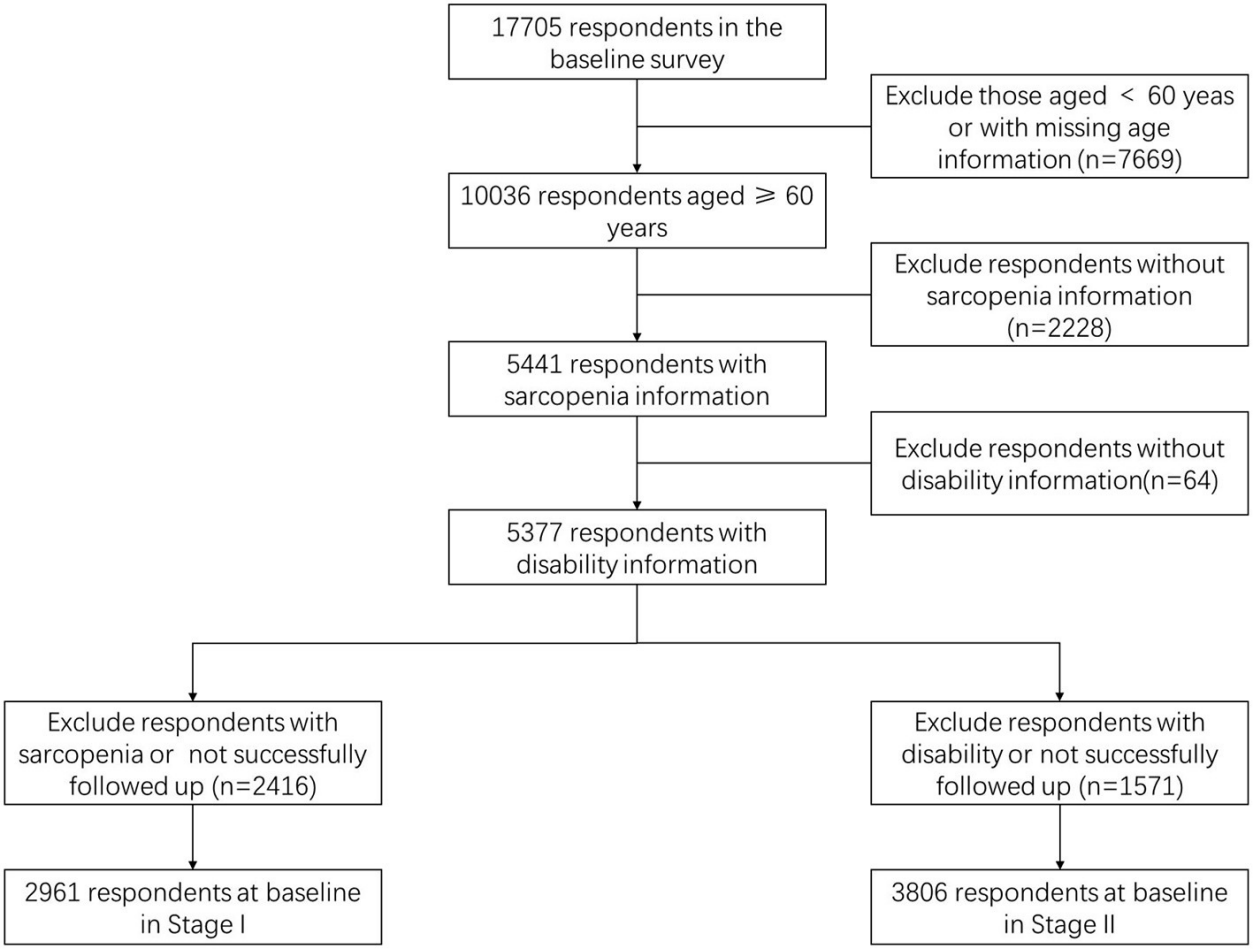
Flowchart of sample screening.

Phase I participants must meet the following requirements: (1) aged ≥60 years, (2) without possible sarcopenia and sarcopenia, (3) collected the information related to ADL, (4) successfully followed up in 2015. A total of 2,961 participants were included in the follow-up analysis (Figure 1). Phase II participants were required to (1) aged ≥60 years, (2) without disability, (3) collected the information related to sarcopenia, (4) successfully followed up in 2015. Ultimately, 3,806 participants were included in the follow-up analysis (Figure 1). Less than 5% of the data were missing covariable information, we imputed missing data based on mean imputation.

### Sarcopenia

In this study, we adopted the AWGS 2019 standard to define and evaluate sarcopenia using three indexes: appendicular skeletal muscle mass (ASM), muscle strength and physical function (20). Sarcopenia is characterized by decreased muscle mass, accompanied by reduced physical function and/or muscle strength. In addition, the 2019 Asian Sarcopenia Working Group pointed out that if older people have normal muscle mass and decreased muscle strength, regardless of whether the physical function is decreased, they are called possible sarcopenia patients.

ASM is calculated using anthropometric equation developed and verified for China population (21). ASM = 0.193 * weight (kg) + 0.107 * height (cm) – 4.157 * sex (male = 1, female = 2) – 0.037 * age – 2.631. Studies have shown strong agreement between dual X-ray absorptiometry (DXA) and the ASM equation model (21, 22). The critical value of muscle mass reduction is determined according to the sex-specific lowest 20% of the height (Ht)-adjusted muscle mass [skeletal muscle mass index (SMI) = ASM/Ht2] within the study population (22–24). In this study, the SMI value of women is < 5.38 kg/m^2^, and that of men is < 7.08 kg/m^2^, which indicates that muscle mass is reduced. Muscle strength was measured using a grip strength meter, and values below 28 kg for males and below 18 kg for females indicate decreased muscle strength. Decline in physical function in this study was defined as a decline in lower limb mobility in the older person, which was assessed using the chair stand test, if an individual takes longer than 12 s to complete the task for five-times, it indicates a decline in physical function.

### Disability

In this study, disability refers to the decreased ability of older people to perform activities of daily living assessed using the basic activities of daily living (BADL) and instrumental activities of daily living (IADL) scales (25). BADLs includes six questions: dressing, getting out of bed, eating, bathing, toileting and continence. IADLs includes five problems: doing housework, taking medication, shopping, cooking and handling finances. Each question includes four answer options: no difficulty; difficult but achievable; some difficulties and need help; unable to complete. The older people are considered to have a disability if they lack complete independence in any question (26, 27).

### Covariates

Other covariates collected included sex (females and males), age, residence (rural and urban), marital status (married/cohabitated and separated/divorced/widowed/never married), educational level (illiterate/primary school, middle school, high school/vocational high school, and junior college or above), smoking status (still smoking, ever smoking, and never smoking), drinking status (drink more than once a month, drink but less than once a month, no drinking), body mass index (BMI < 18.5, 18.5 ≤ BMI < 24, BMI ≥ 24), and annual household expenditure level (in tertiles), whether accompanied by other chronic diseases [high blood sugar (HBS)/diabetes, lung disease, hypertension, heart disease, cancer, stroke, dyslipidemia, digestive disease, kidney disease, liver disease, emotional, nervous, or psychiatric problems, memory-related disease, arthritis or rheumatism and asthma].

### Statistical analyses

We conducted statistical analyses to assess the normality of continuous variables using Skewness-Kurtosis tests. Since the age did not follow a normal distribution (*P* < 0.05), its description was based on the median (p25–p75), and the comparison of baseline characteristics between groups utilized the Wilcoxon rank sum test and Kruskal-Wallis rank sum test. Categorical variables were described with frequency (percentage), and the differences in baseline characteristics between groups were compared using the chi-square test. In phase I, we utilized binary logistic regression models to estimate the odds ratio (ORs) and confidence intervals (CIs) between baseline disability and subsequent sarcopenia. In phase II, binary logistic regression models were also used to investigate whether baseline sarcopenia was associated with an increased risk of disability in older people. Both models were adjusted for potential confounding factors (adjusted Model 1 adjusted for gender and age; adjusted model 2 adjusted all covariates in this study). Finally, sex and age (< 80, ≥80) were analyzed in subgroups. In order to explore whether other chronic diseases will affect the results, we conducted a sensitivity analysis. In addition, we performed additional *post-hoc* power analysis for each of the two phases. Statistical analyses were carried out using STATA version 17 software, with the significance level set at 0.05.

## Results

### Baseline characteristics of the participants

The results showed that in phase I, out of 2,961 participants, 387 (13.07%) had disability, while in phase II, out of 3,806 older persons, 337 (8.85%) participants had possible sarcopenia, 294 (7.72%) had sarcopenia, and 64 (1.68%) had severe sarcopenia. Table 1 presents the baseline characteristics of the two phases.

**Table 1:** Characteristics of the participants.

**Characteristics**		**Phase I (*****n*** = **2,961)**^**a**^			**Phase II (*****n*** = **3,806)**^**b**^				
		**No disability**	**Disability**	***P*** **-value**	**No sarcopenia**	**Possible sarcopenia**	**Sarcopenia**	**Severe sarcopenia**	***P*** **-value**
Number of participants		2,574	387	-	3,111	337	294	64	-
Sex *n* (%)	Male	1,396 (54.23)	156 (40.31)	< 0.001	1,664 (53.49)	171 (50.74)	126 (42.86)	39 (60.94)	0.002
	Female	1,178 (45.77)	231 (59.69)		1,447 (46.51)	166 (49.26)	168 (57.14)	25 (39.06)	
Age		65 (62–69)	66 (62–70)	0.0092	65 (62–69)	67 (63–73)	71 (66–76)	75 (70–78.5)	0.0001
Residence *n* (%)	Rural	2,119 (82.32)	352 (90.96)	< 0.001	2,485 (79.88)	285 (84.57)	268 (91.16)	60 (93.75)	< 0.001
	Urban	455 (17.68)	35 (9.04)		626 (20.12)	52 (15.43)	26 (8.84)	4 (6.25)	
Marital status *n* (%)	Married/cohabitated	412 (16.01)	336 (86.82)	0.153	511 (16.43)	84 (24.93)	90 (30.61)	22 (34.38)	< 0.001
	Separated/divorced/widowed/never married	2,162 (83.99)	51 (13.18)		2,600 (83.57)	253 (75.07)	204 (69.39)	42 (65.63)	
Educational level *n* (%)	Illiterate/primary school	2,055 (79.87)	344 (88.89)	< 0.001	2,464 (79.23)	297 (88.13)	263 (90.07)	58 (90.63)	< 0.001
	Middle school	366 (14.22)	33 (8.53)		443 (14.24)	29 (8.61)	22 (7.53)	4 (6.25)	
	High school/vocational high school	106 (4.12)	9 (2.33)		144 (4.63)	10 (2.97)	7 (2.40)	2 (3.13)	
	Junior college or above	46 (1.79)	1 (0.26)		59 (1.90)	1 (0.30)	0 (0.00)	0 (0.00)	
Smoking status *n* (%)	Still smoking	886 (34.42)	103 (26.61)	0.008	1,043 (33.53)	99 (29.38)	99 (33.67)	19 (29.69)	0.008
	Ever smoking	276 (10.72)	51 (13.18)		344 (11.06)	36 (10.68)	15 (5.10)	12 (18.75)	
	Never smoking	1,412 (54.86)	233 (60.21)		1,724 (55.42)	202 (59.94)	180 (61.22)	33 (51.56)	
Drinking status *n* (%)	Drink more than once a month	693 (26.92)	82 (21.19)	0.027	818 (26.29)	75 (22.26)	70 (23.81)	19 (29.69)	0.079
	Drink but Less than once a month	182 (7.07)	23 (5.94)		227 (7.30)	22 (6.53)	10 (3.40)	4 (6.25)	
	No drinking	1,699 (66.01)	282 (72.87)		2,066 (66.41)	240 (71.22)	214 (72.79)	41 (64.06)	
BMI *n* (%)	< 18.5	146 (5.67)	23 (5.94)	0.193	180 (5.79)	1 (0.30)	147 (50)	25 (39.06)	< 0.001
	18.5–24	1,455 (56.53)	200 (51.68)		1,719 (55.26)	219 (64.99)	147 (50)	39 (60.94)	
	≥24	973 (37.80)	164 (42.38)		1,212 (38.96)	117 (34.72)	0 (0.00)	0 (0.00)	
Household expenditure *n* (%)	Tertile 1	591 (23.94)	67 (18.06)	0.015	829 (26.65)	101 (29.97)	127 (43.20)	29 (45.31)	< 0.001
	Tertile 2	1,250 (50.63)	215 (57.95)		1,507 (48.44)	158 (46.88)	124 (42.18)	21 (32.81)	
	Tertile 3	628 (25.44)	89 (23.99)		775 (24.91)	78 (23.15)	43 (14.63)	14 (21.88)	
High Blood Sugar (HBS)/diabetes *n* (%)	Yes	147 (5.71)	37 (9.56)	0.003	184 (5.91)	20 (5.93)	9 (3.06)	2 (3.13)	0.178
	No	2,427 (94.29)	350 (90.44)		2,927 (94.09)	317 (94.07)	285 (96.94)	62 (96.88)	
Lung disease *n* (%)	Yes	298 (11.58)	69 (17.83)	0.001	356 (11.44)	44 (13.06)	44 (14.97)	10 (15.63)	0.211
	No	2,276 (88.42)	318 (82.17)		2,755 (88.56)	293 (86.94)	250 (85.03)	54 (84.38)	
Hypertension *n* (%)	Yes	737 (28.63)	140 (36.18)	0.002	893 (28.70)	99 (29.38)	51 (17.35)	10 (15.63)	< 0.001
	No	1,837 (71.37)	247 (63.82)		2,218 (71.30)	238 (70.62)	243 (82.65)	54 (84.38)	
Heart disease *n* (%)	Yes	326 (12.67)	79 (20.41)	< 0.001	414 (13.31)	40 (11.87)	31 (10.54)	7 (10.94)	0.486
	No	2,248 (87.33)	308 (79.59)		2,697 (86.69)	297 (88.13)	263 (89.46)	57 (89.06)	
Cancer *n* (%)	Yes	18 (0.70)	5 (1.29)	0.216	20 (0.64)	2 (0.59)	4 (1.36)	1 (1.56)	0.442
	No	2,556 (99.30)	382 (98.71)		3,091 (99.36)	335 (99.41)	290 (98.64)	63 (98.44)	
Stroke *n* (%)	Yes	48 (1.86)	15 (3.88)	0.011	59 (1.90)	9 (2.67)	4 (1.36)	3 (4.69)	0.266
	No	2,526 (98.14)	372 (96.12)		3,052 (98.10)	328 (97.33)	290 (98.64)	61 (95.31)	
Dyslipidemia *n* (%)	Yes	254 (9.87)	49 (12.66)	0.091	311 (10.00)	24 (7.12)	7 (2.38)	3 (4.69)	< 0.001
	No	2,320 (90.13)	338 (87.34)		2,800 (90.00)	313 (92.88)	287 (97.62)	61 (95.31)	
Digestive disease *n* (%)	Yes	555 (21.56)	113 (29.20)	0.001	674 (21.67)	71 (21.07)	67 (22.79)	19 (29.69)	0.451
	No	2,019 (78.44)	274 (70.80)		2,437 (78.33)	266 (78.93)	227 (77.21)	45 (70.31)	
Kidney disease *n* (%)	Yes	150 (5.83)	41 (10.59)	< 0.001	179 (5.75)	27 (8.01)	10 (3.40)	3 (4.69)	0.098
	No	2,424 (94.17)	346 (89.41)		2,932 (94.25)	310 (91.99)	284 (96.60)	61 (95.31)	
Liver disease *n* (%)	Yes	96 (3.73)	23 (5.94)	0.039	127 (4.08)	10 (2.97)	8 (2.72)	1 (1.56)	0.378
	No	2,478 (96.27)	364 (94.06)		2,984 (95.92)	327 (97.03)	286 (97.28)	63 (98.44)	
Emotional, nervous, or psychiatric problems *n* (%)	Yes	28 (1.09)	14 (3.62)	< 0.001	36 (1.16)	3 (0.89)	1 (0.34)	1 (1.56)	0.585
	No	2,546 (98.91)	373 (96.38)		3,075 (98.84)	334 (99.11)	293 (99.66)	63 (98.44)	
Memory-related disease *n* (%)	Yes	37 (1.44)	15 (3.88)	0.001	41 (1.32)	5 (1.48)	8 (2.72)	1 (1.56)	0.293
	No	2,537 (98.56)	372 (96.12)		3,070 (98.68)	332 (98.52)	286 (97.28)	63 (98.44)	
Arthritis or rheumatism *n* (%)	Yes	873 (33.92)	202 (52.20)	< 0.001	1,078 (34.65)	143 (42.43)	106 (36.05)	21 (32.81)	0.040
	No	1,701 (66.08)	185 (47.80)		2,033 (65.35)	194 (57.57)	188 (63.95)	43 (67.19)	
Asthma *n* (%)	Yes	117 (4.55)	31 (8.01)	0.004	135 (4.34)	13 (3.86)	15 (5.10)	2 (3.13)	0.843
	No	2,457 (95.45)	356 (91.99)		2,976 (95.66)	324 (96.14)	279 (94.90)	62 (96.88)	

BMI, body mass index.

Tertile 1, High level of household expenditure; Tertile 2, Medium level of household expenditure; Tertile 3, Low level of household expenditure.

^a^Missing data: 1 for educational level, 121 for household expenditure.

^b^Missing data: 3 for educational level.

### Phase I: the relationship between baseline disability and follow-up sarcopenia

During the 4-year follow-up, 65 (16.8%) new cases of possible sarcopenia, 18 (4.7%) new cases of sarcopenia, and 9 (2.3%) new cases of severe sarcopenia were reported in disabled patients, additional information is shown in Table 2. Those considered disabled had a higher risk of subsequent sarcopenia (crude OR = 1.61; 95% CI = 1.25–2.07). After adjusting for all covariates in 2011, the OR (95% CI) values for older people with disability was 1.35 (1.02–1.79) compared with individuals without disability (Table 3). Subgroup analyses showed that participants with disability aged < 80 years had a higher risk of sarcopenia (OR = 1.42, 95% CI: 1.07–1.89), but the risk of sarcopenia did not differ significantly between sex subgroups (Supplementary Table 1). In addition, as a sensitivity analyses, we excluded patients with concomitant comorbidities of other chronic diseases, and the results did not change substantially, suggesting that the relationship between sarcopenia and disability is unlikely to be influenced by these diseases (Supplementary Table 2). After *post-hoc* power analysis, we found that with the group size, at alpha=0.5 the expected power to detect the difference seen is 96%. If we look for a detectable difference between no sarcopenia, sarcopenia and possible or severe sarcopenia, the power is 95%.

**Table 2:** New case of sarcopenia in phase I.

	**Total**	**Possible sarcopenia**	**Sarcopenia**	**Severe sarcopenia**	**No sarcopenia**
		***n*** **(%)**	***n*** **(%)**	***n*** **(%)**	***n*** **(%)**
Disability	387	65 (16.8)	18 (4.7)	9 (2.3)	295 (76.2)
No disability	2,574	282 (11.0)	97 (3.8)	35 (1.4)	2,160 (83.9)
Total	3,961	347	115	44	2,455

**Table 3:** Logistic regression of disability for sarcopenia.

**Disability**	**Crude**		**Adjusted 1**		**Adjusted 2**	
	**OR (95% CI)**	***P*** **-value**	**OR (95% CI)**	***P*** **-value**	**OR (95% CI)**	***P*** **-value**
No	1 (reference)	-	1 (reference)	-	1 (reference)	-
Yes	1.61 (1.25–2.07)	< 0.001	1.45 (1.12–1.88)	0.006	1.35 (1.02–1.79)	0.038

OR, odds ratio; 95% CI, 95% confidence interval.

Adjusted 1 adjusted for sex, age.

Adjusted 2 adjusted for sex, age, residence, marital status, educational level, smoking status, drinking status, body mass index, and annual household expenditure level, whether accompanied by other chronic diseases [high blood sugar (HBS)/diabetes, lung disease, hypertension, heart disease, cancer, stroke, dyslipidemia, digestive disease, kidney disease, liver disease, emotional, nervous, or psychiatric problems, memory-related disease, arthritis or rheumatism and asthma].

### Phase II: association of baseline sarcopenia with follow-up disability

At this phase, 114 patients with possible sarcopenia, 85 patients with sarcopenia, 23 patients with severe sarcopenia, and 501 individuals with no sarcopenia showed symptoms of disability (Table 4). Compared to patients without sarcopenia, the OR (95% CI) for disability was 2.66 (2.08–3.40) for patients with possible sarcopenia, 2.12 (1.62–2.77) for patients with sarcopenia, and 2.92 (1.74–4.91) for patients with severe sarcopenia. After adjusting for all covariates at baseline, the OR (95% CI) in patients with possible sarcopenia compared with patients without sarcopenia was 2.21 (1.70–2.85), for patients with sarcopenia was 1.58 (1.14–2.19), and for patients with severe sarcopenia, the OR (95% CI) was 1.99 (1.14–3.49; Table 5). Subgroup analysis shows older female patients who may have sarcopenia are at higher risk of disability than men (OR = 2.80, 95% CI: 1.98–3.97), and patients with sarcopenia or severe sarcopenia aged < 80 years had a higher risk of disability (OR = 2.13, 95% CI: 1.52–2.98; OR = 2.98, 95% CI: 1.60–5.54; Supplementary Table 3). In addition, the results did not change after sensitivity analyses (Supplementary Table 4). *Post-hoc* power analysis shows that with the group size, at alpha = 0.05 the expected power to detect the difference seen is 100%.

**Table 4:** New case of disability in phase II.

	**Total**	**Disability**	**No disability**
		***n*** **(%)**	***n*** **(%)**
Possible sarcopenia	337	114 (33.8)	223 (66.2)
Sarcopenia	294	85 (28.9)	209 (71.1)
Severe sarcopenia	64	23 (35.9)	41 (64.1)
No sarcopenia	3,111	501 (16.1)	2,610 (83.9)
Total	3,806	723	3,083

**Table 5:** Logistic regression of sarcopenia for the odds of disability.

**Sarcopenia**	**Crude**		**Adjusted 1**		**Adjusted 2**	
	**OR (95% CI)**	***P*** **-value**	**OR (95% CI)**	***P*** **-value**	**OR (95% CI)**	***P*** **-value**
No	1 (reference)	-	1 (reference)	-	1 (reference)	-
Possible sarcopenia	2.66 (2.08–3.40)	< 0.001	2.34 (1.82–3.01)	< 0.001	2.21 (1.70-2.85)	< 0.001
Sarcopenia	2.12 (1.62–2.77)	< 0.001	1.45 (1.09–1.94)	0.010	1.58 (1.14-2.19)	0.006
Severe sarcopenia	2.92 (1.74–4.91)	< 0.001	1.86 (1.09–3.20)	0.024	1.99 (1.14-3.49)	0.016

OR, odds ratio; 95% CI, 95% confidence interval.

## Discussion

In our study, we observed a bidirectional relationship between disability and sarcopenia. Specifically, disability in older people increases the risk of developing sarcopenia, while possible sarcopenia, sarcopenia, and severe sarcopenia also increase the risk of subsequent disability. Even after adjusting for sex, age, or other confounders, the relation still existed. Furthermore, the connection between disability and sarcopenia exhibited some variation in subgroup analyses based on age and sex.

Several scholars in the field have analyzed the impact of sarcopenia on disability. For instance, Phillips et al. noted that sarcopenia results in higher disability scores in older people, and the 3-year incidence of disability was ~32.7% (28). Moreover, in a cross-sectional analyses of 27,924 participants in the Canadian Longitudinal Study (29), sarcopenia was associated with an increased risk of ADL disability. It is worth noting that studies have indicated that older individuals with sarcopenia exhibit lower levels of basic and instrumental activities of daily living compared to those without sarcopenia (30, 31), suggesting that both may be influenced by sarcopenia.

Sarcopenia as a risk factor for subsequent disability is confirmed by the fact that sarcopenia is associated with future disability, even after adjusting for sex, age, and other covariates. Proactively preventing and managing sarcopenia has been shown to effectively reduce the risk of disability (32, 33), therefore, it is recommended that sarcopenia should be included when screening for disability. Additionally, our study found that the ORs for increased risk of disability did not progressively increase by severity of sarcopenia. Patients with possible sarcopenia and severe sarcopenia displayed a higher risk of disability, while patients with sarcopenia had a relatively lower risk, it is an interesting phenomenon. This may because that a higher number of patients with possible sarcopenia had reduced physical function and therefore a higher risk of subsequent disability, whereas all patients with severe sarcopenia had reduced physical function. Therefore, patients with possible sarcopenia (especially with reduced physical function) need to be given equivalent attention as patients with severe sarcopenia when it comes to preventing disability in the older person. We are currently unaware of studies investigating the impact of disability on sarcopenia. In our study, disability remained positively associated with subsequent sarcopenia even after adjustment for covariables. Older people who are impaired in physical activity (34) and spend most of their time in a sedentary state (35) are at increased risk for sarcopenia, which may contribute to the results of the study.

Several explanations may elucidate the bidirectional relation between disability and sarcopenia. Firstly, sarcopenia may lead to an increased number of falls (6) and reduced exercise participation (36) in older people, consequently increasing the risk of disability. Similarly, decreased mobility (37) and heightened risk of malnutrition (38) in disabled older people may also contribute to the onset of sarcopenia. If the energy intake is low and cannot match the energy expenditure level, it will lead to weight loss and loss of muscle mass in the older people. In addition, as the amount of food consumed by the older people decreases, it may lead to difficulties in meeting nutritional needs, especially micronutrients, which will also increase the risk of sarcopenia in the older people (39). Secondly, aging will leads to heightened inflammation levels, which directly impacts the metabolism of muscle tissue and bone (40), ultimately causing declining physical function or disability. Elevated inflammation levels may play a role in the bidirectional correlation between sarcopenia and disability in the older people. One study has indicated (41) that increased levels of superoxide dismutase (SOD), the main antioxidant enzyme, reduce the risk of disability in older individuals. Moreover, higher levels of oxidative stress are associated with an increased risk of sarcopenia, indicating that oxidative stress levels may influence the relationship between sarcopenia and disability (42). Finally, it is important to note that older individuals with chronic diseases, such as diabetes (43) and COPD (44), are at an increased risk of developing sarcopenia. Similarly, diabetes (45) and COPD (46) can elevate the risk of disability.

In the subgroup analyses, we found a higher risk of subsequent sarcopenia in disabled persons aged < 80 years, as well as a higher prevalence of disability in individuals aged < 80 years with sarcopenia and severe sarcopenia. This could be attributed to the higher occurrence of malnutrition, reduced physical activity, and decreased physical function in older individuals aged ≥80 years, thereby weakening the relationship between the two conditions. Consequently, in the prevention of sarcopenia and disability, greater attention should be directed toward disabled or sarcopenia patients < 80 years of age, and all older people ≥80 years of age. Furthermore, in a subgroup analysis by sex, women with possible sarcopenia are more susceptible to disability than men. This may be due to women having less time for physical activity (47) and poorer health status (48) compared to men, and the allocation of social and family roles that negatively affects their access to healthcare and health protection based on traditional Chinese cultural beliefs. Additionally, previous studies have also indicated that older women are more severely disabled than men (49, 50), which could be related to the above reasons. Taken together, our study results advocate for the consideration of sex and age effects when formulating intervention strategies for sarcopenia or disability.

The study used a nationally representative cohort survey to reflect the general health status of Chinese older adults, it has large sample size and a long follow-up period. Second, it may be the first study to examine the bidirectional relationship between disability and sarcopenia using a single cohort. In addition, this study adjusted for confounding variables including gender, age, education level, and other baseline characteristics. However, we should also note the limitations of this study. First, some disease-related data were self-reported, and these diseases may generate measurement errors. In addition, there may be other unmeasured confounders influencing the association between disability and sarcopenia, but it is difficult to avoid this issue in most observational studies. Finally, the follow-up interval in this study was 4 years, and future studies need to conduct longer follow-ups to analyze whether the bidirectional association between disability and sarcopenia can be sustained over a longer period of time.

## Conclusions

In conclusion, we found a bidirectional relation between disability and sarcopenia. Disability can influence subsequent sarcopenia, and sarcopenia can also predict the incidence of subsequent disability. Screening and timely management of sarcopenia should be enhanced to prevent disability in older people. Furthermore, when assessing the relationship between disability and sarcopenia, we should be mindful of the impact of gender and age to help clinical staff develop more targeted and applicable interventions to promote healthy aging.

## Data availability statement

The original contributions presented in the study are included in the article/Supplementary material, further inquiries can be directed to the corresponding authors.

## Ethics statement

The studies involving humans were approved by Peking University Biomedical Ethics Review Committee (IRB00001052-11015). The studies were conducted in accordance with the local legislation and institutional requirements. Written informed consent for participation was not required from the participants or the participants' legal guardians/next of kin in accordance with the national legislation and institutional requirements.

## Author contributions

LL: Writing – review & editing, Writing – original draft, Conceptualization. YZ: Writing – review & editing, Writing – original draft, Validation. YS: Methodology, Software, Writing – original draft, Writing – review & editing. LW: Writing – original draft, Methodology. LM: Writing – review & editing, Software. TZ: Writing – review & editing, Investigation.
