# Supplementary material for: The bidirectional relationship between sarcopenia and disability in China: a longitudinal study from CHARLS
Source: Front Public Health. 2024 May 7;12:1309673. doi: 10.3389/fpubh.2024.1309673 (PMC11106476; doi:10.3389/fpubh.2024.1309673)
Supplement: Supplementary file 1 [file Table_1.docx]

Supplementary Table S1 Odds ratios of disability for sarcopenia in sub-group analyses

|  | Crude | | Adjusted 2 | |
| --- | --- | --- | --- | --- |
|  | OR (95%CI) | *P* value | OR (95%CI) | *P* value |
| male |  |  |  |  |
| No disability | 1(reference) | - | 1(reference) | - |
| Disability | 1.76(1.20-2.59) | 0.004 | 1.28(0.82-1.99) | 0.277 |
| female |  |  |  |  |
| No disability | 1(reference) | - | 1(reference) | - |
| Disability | 1.48(1.05-2.08) | 0.025 | 1.45(0.99-2.10) | 0.052 |
| ＜80 |  |  |  |  |
| No disability | 1(reference) | - | 1(reference) | - |
| Disability | 1.52(1.17-1.98) | 0.002 | 1.42(1.07-1. 89) | 0.017 |
| ≥80 |  |  |  |  |
| No disability | 1(reference) | - | 1(reference) | - |
| Disability | 1.73(0.58-5.11) | 0.323 | 0.52(0.03-9.69) | 0.662 |

OR, odds ratio; 95% CI, 95% confidence interval.

Supplementary Table S2 Sensitivity analyses on the relation between sarcopenia and disability（Phase I）

| Excluding diseases | Phase I (Disability vs. No disability) | |
| --- | --- | --- |
|  | Adjusted OR (95%CI) | *P* value |
| High Blood Sugar (HBS)/Diabetes | 1.32(1.00-1.76) | 0.050 |
| Lung disease | 1.36(1.02-1.80) | 0.034 |
| Hypertension | 1.35(1.02-1.79) | 0.036 |
| Heart disease | 1.36(1.03-1.81) | 0.031 |
| Cancer | 1.35(1.02-1.79) | 0.037 |
| Stroke | 1.34(1.01-1.78) | 0.040 |
| Dyslipidemia | 1.35(1.02-1.79) | 0.039 |
| Digestive disease | 1.35(1.02-1.79) | 0.036 |
| Kidney disease | 1.34(1.01-1.78) | 0.041 |
| Liver disease | 1.35(1.02-1.79) | 0.037 |
| Emotional, nervous, or psychiatric problems | 1.35(1.02-1.80) | 0.035 |
| Memory-related disease | 1.34(1.01-1.78) | 0.040 |
| Arthritis or Rheumatism | 1.33(1.00-1.76) | 0.047 |
| Asthma | 1.35(1.02-1.79) | 0.038 |

OR, odds ratio; 95% CI, 95% confidence interval.

The multivariable models adjusted for the following variables: sex, age, residence, marital status, educational level, smoking status, drinking status, body mass index, annual household expenditure level, whether accompanied by other chronic diseases (high blood sugar (HBS)/diabetes, lung disease, hypertension, heart disease, cancer, stroke, dyslipidemia, digestive disease, kidney disease, liver disease, emotional, nervous, or psychiatric problems, memory-related disease, arthritis or rheumatism and asthma).

Supplementary Table S3 Odds ratios of sarcopenia for disability in sub-group analyses

|  | Crude | | Adjusted 2 | |
| --- | --- | --- | --- | --- |
|  | OR (95%CI) | *P* value | OR (95%CI) | *P* value |
| male |  |  |  |  |
| No sarcopenia | 1(reference) | - | 1(reference) | - |
| Possible Sarcopenia | 2.20(1.53-3.18) | ＜0.001 | 1.71(1.15-2.53) | 0.008 |
| Sarcopenia | 1.85(1.19-2.86) | 0.006 | 1.38(0.82-2.32) | 0.225 |
| Severe Sarcopenia | 2.74(1.37-5.49) | 0.004 | 1.52(0.71-3.24) | 0.276 |
| female |  |  |  |  |
| No sarcopenia | 1(reference) | - | 1(reference) | - |
| Possible Sarcopenia | 3.12(2.23-4.36) | ＜0.001 | 2.80(1.98-3.97) | ＜0.001 |
| Sarcopenia | 2.19(1.55-3.10) | ＜0.001 | 1.83(1.19-2.80) | 0.006 |
| Severe Sarcopenia | 3.44(1.54-7.66) | 0.002 | 2.66(1.12-6.29) | 0.026 |
| ＜80 |  |  |  |  |
| No sarcopenia | 1(reference) | - | 1(reference) | - |
| Possible Sarcopenia | 2.59(2.01-3.34) | ＜0.001 | 2.39(1.84-3.11) | ＜0.001 |
| Sarcopenia | 2.10(1.57-2.80) | ＜0.001 | 2.13(1.52-2.98) | ＜0.001 |
| Severe Sarcopenia | 2.76(1.52-4.99) | 0.001 | 2.98 (1.60-5.54) | 0.001 |
| ≥80 |  |  |  |  |
| No sarcopenia | 1(reference) | - | 1(reference) | - |
| Possible Sarcopenia | 2.38(0.82-6.94) | 0.112 | 3.82 (0.99-14.79) | 0.053 |
| Sarcopenia | 0.99(0.42-2.33) | 0.982 | 1.03(0.30-3.61) | 0.960 |
| Severe Sarcopenia | 1.43(0.44-4.66) | 0.555 | 1.98(0.35-11.11) | 0.437 |

OR, odds ratio; 95% CI, 95% confidence interval.

Supplementary Table S4 Sensitivity analyses on the relationship between sarcopenia and subsequent disability (Phase II)

| Excluding diseases | Phase II | | | | | |
| --- | --- | --- | --- | --- | --- | --- |
|  | Possible sarcopenia | | Sarcopenia | | Severe possible | |
|  | Adjusted OR (95%CI) | *P* value | Adjusted OR (95%CI) | *P* value | Adjusted OR (95%CI) | *P* value |
| High Blood Sugar (HBS)/Diabetes | 2.21(1.71-2.86) | ＜0.001 | 1.59(1.15-2.20) | 0.005 | 2.00(1.14-3.50) | 0.015 |
| Lung disease | 2.22(1.71-2.87) | ＜0.001 | 1.58(1.14-2.19) | 0.006 | 2.00(1.14-3.50) | 0.016 |
| Hypertension | 2.20(1.70-2.85) | ＜0.001 | 1.58(1.14-2.19) | 0.006 | 1.98(1.13-3.47) | 0.016 |
| Heart disease | 2.19(1.69-2.83) | ＜0.001 | 1.58(1.14-2.19) | 0.006 | 2.00(1.14-3.49) | 0.015 |
| Cancer | 2.21(1.71-2.85) | ＜0.001 | 1.58(1.14-2.19) | 0.006 | 1.99(1.13-3.47) | 0.016 |
| Stroke | 2.20(1.70-2.85) | ＜0.001 | 1.58(1.14-2.19) | 0.006 | 1.98(1.13-3.47) | 0.016 |
| Dyslipidemia | 2.20(1.70-2.85) | ＜0.001 | 1.58(1.14-2.19) | 0.006 | 1.99(1.14-3.49) | 0.016 |
| Digestive disease | 2.21(1.70-2.85) | ＜0.001 | 1.58(1.14-2.19) | 0.006 | 1.99(1.14-3.49) | 0.016 |
| Kidney disease | 2.21(1.71-2.86) | ＜0.001 | 1.58(1.14-2.18) | 0.006 | 1.99(1.14-3.48) | 0.016 |
| Liver disease | 2.20(1.70-2.84) | ＜0.001 | 1.58(1.14-2.18) | 0.006 | 1.98(1.13-3.46) | 0.017 |
| Emotional, nervous, or psychiatric problems | 2.20(1.70-2.85) | ＜0.001 | 1.58(1.14-2.18) | 0.006 | 2.00(1.14-3.50) | 0.015 |
| Memory-related disease | 2.20(1.70-2.85) | ＜0.001 | 1.59(1.15-2.21) | 0.005 | 1.98(1.14-3.47) | 0.016 |
| Arthritis or Rheumatism | 2.24(1.73-2.90) | ＜0.001 | 1.59(1.15-2.20) | 0.005 | 1.98(1.13-3.47) | 0.017 |
| Asthma | 2.20(1.70-2.84) | ＜0.001 | 1.58(1.14-2.19) | 0.006 | 1.97(1.12-3.44) | 0.018 |

OR, odds ratio; 95% CI, 95% confidence interval.

The multivariable models adjusted the following variables: sex, age, residence, marital status, educational level, smoking status, drinking status, body mass index, annual household expenditure level, whether accompanied by other chronic diseases (high blood sugar (HBS)/diabetes, lung disease, hypertension, heart disease, cancer, stroke, dyslipidemia, digestive disease, kidney disease, liver disease, emotional, nervous, or psychiatric problems, memory-related disease, arthritis or rheumatism and asthma).
